# Overexpression of Human and Fly Frataxins in Drosophila Provokes Deleterious Effects at Biochemical, Physiological and Developmental Levels

**DOI:** 10.1371/journal.pone.0021017

**Published:** 2011-07-11

**Authors:** Juan A. Navarro, José V. Llorens, Sirena Soriano, José A. Botella, Stephan Schneuwly, María J. Martínez-Sebastián, María D. Moltó

**Affiliations:** 1 Institute of Zoology, University of Regensburg, Regensburg, Germany; 2 Departament de Genètica, Universitat de València, Burjassot, Valencia, Spain; 3 Instituto de Biomedicina, CSIC, Valencia, Spain; 4 CIBERSAM (Centro de Investigación Biomédica en Red de Salud Mental), Madrid, Spain; Brigham and Women's Hospital, Harvard Medical School, United States of America

## Abstract

**Background:**

Friedreich's ataxia (FA), the most frequent form of inherited ataxias in the Caucasian population, is caused by a reduced expression of frataxin, a highly conserved protein. Model organisms have contributed greatly in the efforts to decipher the function of frataxin; however, the precise function of this protein remains elusive. Overexpression studies are a useful approach to investigate the mechanistic actions of frataxin; however, the existing literature reports contradictory results. To further investigate the effect of frataxin overexpression, we analyzed the consequences of overexpressing human (FXN) and fly (FH) frataxins in *Drosophila.*

**Methodology/Principal Findings:**

We obtained transgenic flies that overexpressed human or fly frataxins in a general pattern and in different tissues using the UAS-GAL4 system. For both frataxins, we observed deleterious effects at the biochemical, histological and behavioral levels. Oxidative stress is a relevant factor in the frataxin overexpression phenotypes. Systemic frataxin overexpression reduces *Drosophila* viability and impairs the normal embryonic development of muscle and the peripheral nervous system. A reduction in the level of aconitase activity and a decrease in the level of NDUF3 were also observed in the transgenic flies that overexpressed frataxin. Frataxin overexpression in the nervous system reduces life span, impairs locomotor ability and causes brain degeneration. Frataxin aggregation and a misfolding of this protein have been shown not to be the mechanism that is responsible for the phenotypes that have been observed. Nevertheless, the expression of human frataxin rescues the aconitase activity in the *fh* knockdown mutant.

**Conclusion/Significance:**

Our results provide *in vivo* evidence of a functional equivalence for human and fly frataxins and indicate that the control of frataxin expression is important for treatments that aim to increase frataxin levels.

## Introduction

Friedreich's ataxia (FA), an autosomal recessive disease, is the most frequent form of inherited ataxias in the Caucasian population (1∶50000) [1]. The major cause of this disease is the presence of a large GAA repeat expansion in the first intron of the *FXN* gene [2]. This large GAA repeat decreases the level of transcription of the mRNA that encodes the protein frataxin [3], [4], resulting in levels that range from 5% to 30% of the normal level of this protein [5]. The clinical manifestations of FA involve spinal cord and cerebellum neurodegeneration, which cause gait and limb ataxia, muscular weakness and speech impairments [6], [7]. Other manifestations of FA include scoliosis, diabetes and hypertrophic cardiomyopathy, which is the main cause of death [8].

Frataxin is a highly conserved protein throughout evolution [9]. This degree of conservation has enabled the development of FA models in many organisms, from *E.coli* to the mouse, that have contributed to a better understanding of this protein's function; however, the exact function of frataxin remains elusive. Seminal findings by a number of key studies have suggested potential roles for frataxin in iron homeostasis [10]–[14], as an activator of the respiratory chain [15]–[17], as a regulator of Fe-S cluster assembly through activation [12], [18]–[23] or inhibition [24] and/or by promoting cellular defense against reactive oxygen species [25]–[32].

In *Drosophila*, the frataxin homolog (*fh*) shares a high degree of sequence conservation and projected folding with other frataxin orthologs [33]. Moreover, a reduction in the level of frataxin expression in *Drosophila* has been established as an effective model to study frataxin function and the pathological mechanisms that underlie frataxin deficiency. In fact, the loss of *fh* recapitulates important behavioral and biochemical features of human disease [31], [34]. Furthermore, *Drosophila* models have provided support for the crucial involvement of oxidative stress, particularly peroxides, in the development of FA [31], [32], [35]. These models have also indicated that frataxin is relevant in glial cells and that these cells play a role in FA [35]. In addition, these models have revealed that mitochondrial depolarization is an initial element in the axonal transport defects that lead to a concomitant dying-back neuropathy [36]. Overexpression studies in the fruit fly have been greatly used to study the gene's function and to provide insight into the human inherited pathologies. Among these studies, several reports of investigations of the ectopic expression of human genes in *Drosophila* have provided highly valuable information regarding Alzheimer's disease [37], polyglutamine diseases [38], Parkinson's disease [39], [40] and dominant spinocerebellar ataxias (SCAs) [41], [42].

For frataxin overexpression, the existing literature presents contradictory results. Experiments in mice [17], [43] or in cultured cells [15], [44], [45] have revealed that frataxin overexpression was innocuous or had a positive effect on the cell's biology, stimulating the production of ATP or inducing the recruitment of antioxidant defenses. Similarly, Runko *et al.* [46] reported that the overexpression of *Drosophila* frataxin promoted cellular resistance to oxidative stress. However, we have previously reported that *Drosophila* frataxin overexpression [31] leads to detrimental phenotypes in the fly, including developmental defects, a decrease in the level of aconitase activity and hypersensitivity to oxidative stress. Notably, the overexpression of frataxin in yeast has also been shown to critically affect aconitase activity [47].

In the present study, we analyzed the effects of the overexpression of two frataxins in a multicellular organism, *Drosophila melanogaster*. To achieve this aim, we generated transgenic flies that overexpressed human (*FXN*) and fly (*fh*) frataxins through the UAS/GAL4 system. We also studied whether FXN can functionally replace endogenous *Drosophila* frataxin. In the present paper, we report that the increased expression of human or fly frataxin in *Drosophila* leads to deleterious effects at biochemical, histological and behavioral levels. We also show that FXN can rescue the reduction in the aconitase activity that is associated with the loss of frataxin in the fly. Our results provide *in vivo* evidence of a functional equivalence between human and fly frataxins and indicate that the regulation of frataxin expression is a key factor that underlies frataxin function.

## Materials and Methods

### Drosophila stocks

The *w* ^1118^ strain of *Drosophila* was used as the control line and for the injection of the UAS-*FXN* construct. The UAS-*fh* line, which carried the *fh* coding sequence under the control of UAS, was previously generated in our laboratory [31]. The UAS-*fh* line induced a 9-fold increase in the level of *fh*-mRNA at 29°C. The *UD1R2* line was kindly provided by J.P. Phillips (University of Guelp, Guelp, ON). *UD1R2* induced a strong interference of *fh*, and the FH protein was reduced to undetectable levels [34]. The MitoCat flies were a gift from W. Orr (Southern Methodist University, Dallas, USA). The *actin*-GAL4, *da* ^G32^-GAL4, *24B*-GAL4, *neur*-GAL4, *repo*-GAL4 and *Appl*-GAL4 driver lines were obtained from the Bloomington Stock Center. The stocks were maintained at 25°C using standard cornmeal agar medium. The crosses between the GAL4 drivers and the UAS responder lines were conducted at either 25°C or 29°C. The rescue experiments were conducted by generating the following stocks: *mitoCat* / CyO; UAS*-FXN* / TM3; and *UDIR2* / CyO; UAS*-FXN* / TM3.

### Construction of the UAS-*FXN* transgene and the generation of the fly transformants

The cDNA for *FXN* was obtained from human fetal brain poli-(A)+ mRNA (Invitrogen). A 645 bp fragment, which included the entire coding region of the gene, was amplified using the following primers: *FXN*-pUASTf (CTCGAGATGTGGACTCTCGGGCGCCG) and *FXN*-pUASTr (GGTACCTCAAGCATCTTTTCCGGAATAGGCCAAG). This fragment was inserted into the pCR2.1-TOPO vector and was then subcloned into the pUAST vector to generate the UAS-*FXN* transgene.

The transgenic flies were generated using standard embryo injection protocols [48]. Seven independent lines were obtained, and the presence of the transgene was verified in each line using PCR with vector-specific primers. The sequencing of the PCR products revealed that there were no mutations present in the *FXN* sequence. The lines were examined for the expression of *FXN* by crossing each of these lines with the *actin*-GAL4 driver line. A line that contained the UAS-*FXN* transgene on the second chromosome was selected to perform our experiments.

### Western blotting

The total protein extraction from the *Drosophila* larvae was performed as previously described [49]. The protein levels were determined using the Bradford assay. The samples were separated on 5% stacking, 15% separating SDS polyacrylamide gels. The resolved proteins were electroblotted to a Hybond-ECL nitrocellulose membrane (GE Healthcare) and were probed using mouse anti-FXN (Chemicon, Millipore, 1∶2000), mouse anti-NDUFS3 (Mitosciences MS112, 1∶2000) or mouse anti-α-tubulin (Sigma-Aldrich, 1∶2500) antibodies. Fluorescent goat anti-mouse was used as the secondary antibody in these cases. Detection and quantification was conducted using the Odyssey system (Li-cor Inc.). Alternatively, goat anti-mouse IgG horseradish peroxidase conjugate (Sigma-Aldrich) was used as a secondary antibody and was detected using ECL Detection Reagent (GE Healthcare).

### Immunohistochemistry staining

The whole mount embryo staining technique with horseradish peroxidase was conducted as previously described [50]. The embryos were incubated with the following primary antibodies: mouse mAb anti-myosin heavy chain (anti-MHC), 1∶8 dilution, a gift from D. Kiehart; mouse mAb 22C10 anti-peripheral nervous system neurons (anti-PNS), 1∶50 dilution; mouse mAb BP102 anti-central nervous system axons (anti-CNS), 1∶200 dilution, from the Developmental Studies Hybridoma Bank; and rabbit anti-even-skipped protein, 1∶2000 dilution, kindly provided by M. Frasch. The Ab-antigen complexes were detected using biotinylated horse anti-mouse IgG (Pierce, Rockford, IL) or biotinylated goat anti-rabbit IgG (Pierce) antibodies.

### Brain histology

For the examination of the adult fly brains using light and electron microscopy, ultrathin Epon plastic sections were post-stained with 2% uranyl acetate, which was followed by Reynolds' lead citrate. Next, the sections were stabilized for transmission electron microscopy using carbon coating. The examination was conducted using a Zeiss EM10C/VR electron microscope at 80 kV. The glial cell material was identified by its characteristically higher electron density.

### Life span determination and climbing assay

For the life span determination, the male flies were collected within 24 h of eclosion and were raised at 25°C under a 12 h∶12 h light/dark cycle. These flies were transferred to fresh food vials every 2–3 days. The climbing assay was conducted as described in Botella *et al.* [51].

### Assay of the aconitase activity

The total aconitase activity was determined from L3 larvae using the Bioxytech Aconitase-340™ Spectrophotometric Assay Kit (Oxis International Inc, Portland, OR).

### Hyperoxia treatment

The hyperoxia treatment was started one day post-eclosion and was performed as previously described [31]. To measure the aconitase activity, L3 larvae were maintained in hyperoxia conditions for a 24 h period before performing the assay.

### Gel filtration chromatography

The mitochondria from the *actin*-GAL4>UAS-*FXN* larvae were isolated (MITOISO1, Sigma), lysed in hypotonic buffer (HEPES 10 mM, pH 7.0) and sonicated (three times for 30 sec) before being centrifuged at 20,000 *g* for 30 min. The mitochondrial matrix proteins were subjected to size exclusion chromatography on a Superdex 200 10/300 GL column with a fractionation range of 10 to 600 kDa (GE Healthcare) and were eluted with 50 mM HEPES and 140 mM NaCl, pH 8.0, at a flow rate of 0.5 ml/min. Blue Dextran 2000 (1 mg/ml) was used to estimate the void volumes, and gel filtration molecular weight standards (GE Healthcare) were used to calibrate the column. An equal volume of each fraction was analyzed using SDS-PAGE and western blotting.

### Statistical analysis

A Kaplan-Meier analysis of the survival data with a semi-parametric log rank test was performed using Graph Pad Prism 4.0 software. The differences in the locomotor and aconitase activities were tested using a one-way ANOVA test, using the Statistical Packages for the Social Sciences (SPSS) v17.0. A value of p<0.05 was considered to be statistically significant.

## Results

### Human frataxin is correctly expressed and targeted to the mitochondria in *Drosophila*

**Figure 1:**
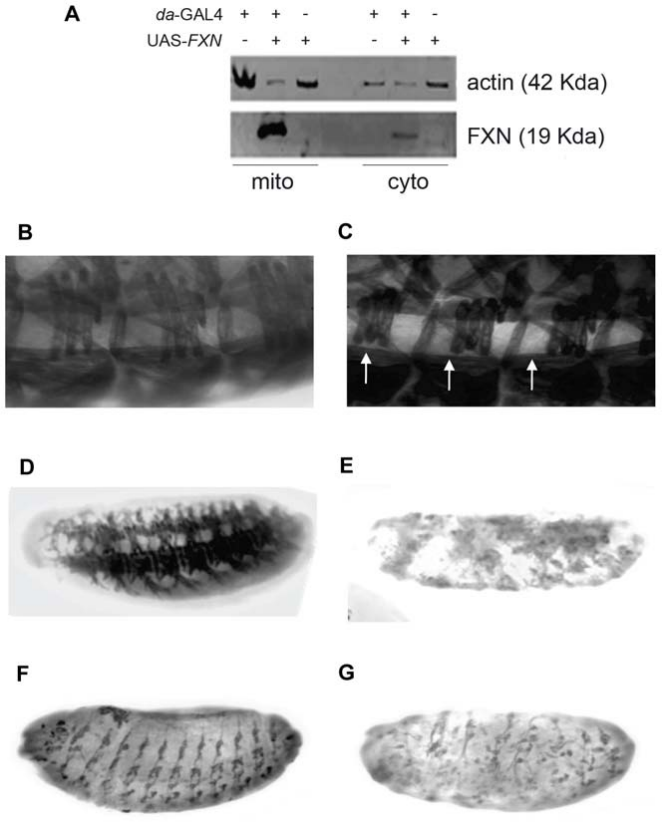
Effect of *FXN* overexpression in the embryonic development. (A) Detection of the FXN protein in *da-*GAL4>UAS-*FXN* (+,+) larvae in the mitochondrial (mito) and the cytosolic (cyto) fractions. The control genotypes of the larvae were *da*-GAL4>*yw* (+,−) and *yw*>UAS-*FXN* (−,+). The human frataxin protein was localized in the mitochondria. Anti-actin was used as a control for cytosolic contamination. (B–G) Muscular and nervous system defects in *da*-GAL4>UAS-*FXN* and *actin*-GAL4>UAS-*FXN* embryos at stage 16. In these panels, anterior is toward the left, and all of the views are lateral views. Anti-myosin staining revealed abnormalities in the junctions of lateral transversal muscles 1, 2 and 3 and the ventral longitudinal muscle 1 (C) compared with the control (B). Moreover, a few embryos exhibited abnormalities in the muscular development of mutant (E) versus control (D) embryos. Staining with 22C10 detected strong abnormalities in the axonal path finding of the sensory nerves (G) with respect to the control (F).

To investigate the effect of human *FXN* expression in *D. melanogaster*, we generated transgenic flies that carried the UAS-*FXN* construct. These flies were crossed with the *da*-GAL4 driver line at 25°C to reach the ubiquitous expression of the human gene. Because the *da-*GAL4>UAS-*FXN* individuals exhibited lethality before adult eclosion, the presence of human frataxin was confirmed in the transgenic larvae using western blotting. As expected, human frataxin was only detected in the *da-*GAL4>UAS-*FXN* larvae; no signal was observed in the driver and responder controls (Figure 1A). To test whether *FXN* was transported into the mitochondria, we analyzed the relative amount of frataxin in the mitochondrial and cytosolic fractions. We used an anti-actin antibody as a control for cytosolic contamination. In three independent experiments, the amount of frataxin was consistently found to be 8-10 times higher in the mitochondrial fraction in comparison to the cytosolic fraction, with only a residual amount of frataxin being present in the cytoplasm. The quantity of actin was similar in both fractions. Therefore, our results indicate that human frataxin is mainly localized within the mitochondria in *Drosophila* cells, as it exhibits the same subcellular localization as endogenous *Drosophila* frataxin [31].

### Human frataxin overexpression reduces *Drosophila* viability

We have previously reported the consequences of increasing the amount of *fh* expression in *Drosophila* [31]. We found that the general and mesodermal overexpression of *fh* at 29°C resulted in lethality during the pre-adult stages and restricted the expression of *fh* in the nervous system, which had no effect on viability.

To test whether the effect of *FXN* overexpression on *Drosophila* viability was similar to the effect of the overexpression of *fh*, we first investigated systemic *FXN* expression with the ubiquitous *da*-GAL4 and *actin*-GAL4 drivers by mating the flies at 25°C and 29°C. Full lethality was observed with both drivers at both temperatures. These results were similar to those after the *fh* overexpression (Table 1); however, deaths were observed earlier in the individuals with *FXN* expression than in those with *fh* overexpression.

**Table 1:** The effect of general and tissue-specific expression of human and fly frataxins on *Drosophila* viability at 25°C and 29°C.

Expression pattern	GAL4 drivers	*FXN* expression	*fh* overexpression
Ubiquitous	*actin*	Lethal	Lethal
	*da*	Lethal	Lethal
Muscular system	*24B*	Lethal	Lethal
Nervous system	*Appl*	Viable at 25°C	Viable
		Lethal at 29°C	
	*neur*	Viable	Viable
	*repo*	Viable	Viable

Next, we examined the consequences of a tissue-specific expression of *FXN*. The *FXN* expression was specifically driven in the nervous system and in the muscles, which are two of the most affected tissues in patients with FA. The *FXN* expression in the embryonic mesoderm (*24B*-GAL4) led to the death of all of the individuals that were early to late pupae. However, we observed viable progeny that expressed *FXN* in the central nervous system (CNS), in the sensory organs and their precursors and in the glial cells, using the *Appl*-GAL4, *neur*-GAL4 and *repo*-GAL4 drivers, respectively. In contrast, the expression of *FXN* at 29°C with the neuronal post-mitotic driver *Appl*-GAL4 resulted in pre-adult lethality (Table 1).

Collectively, these results demonstrate that tissues respond similarly to *Drosophila* and human frataxin overexpression and that an adequate balance of the systemic synthesis of frataxin is critical for fly viability.

### The general expression of *FXN* disrupts normal *Drosophila* development

Inmunohistochemical staining was conducted in *da*-GAL4>UAS-*FXN* and *actin*-GAL4>UAS-*FXN* embryos to identify the underlying defects that were associated with the lethal phenotypes. Anti-myosin staining of the muscular system revealed defects in the junctions of lateral transversal muscles 1, 2 and 3 with ventral longitudinal muscle 1, which was likely due to deficient muscle growth (Figure 1C). Defects in several muscles were also reported after *fh* overexpression [31]. In a few cases, we observed *FXN*-expressing embryos that exhibited a disrupted muscular system (Figure 1E). After the embryo's PNSs were stained using the 22C10 antibody, a strong disorganization of the sensory axons was detected (Figure 1G). Similar alterations were described for the *da*-GAL4 driven overexpression of *fh* [31]. No abnormalities were found in the CNS of the *FXN*-expressing embryos when they were stained using the BP102 antibody (as in *da*-GAL4>UAS-*fh* embryos; data not shown).

We observed that the lethal phenotypes that were associated with the general overexpression of human or fly frataxin mainly resulted from the impairment of correct muscle and PNS development, whereas the CNS was not affected. The high degree of similarity between the defects that were observed with the overexpression of human and fly frataxins supports the involvement of the overexpression of FXN in the same developmental mechanisms than FH overexpression. In addition, these results indicate that the frataxin level is critical for the normal embryonic development of muscle and the PNS.

### Nervous system expression of *FXN* shortens life span, impairs locomotor performance and causes brain degeneration

**Figure 2:**
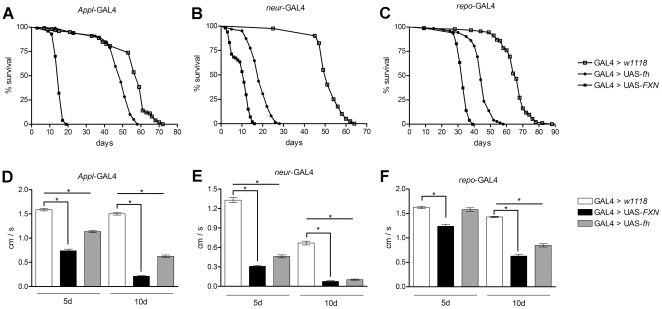
Physiological and behavioral defects induced by frataxin overexpression in nervous system. (A–C) Life span under normoxia conditions. Overexpression of human (black square) or *Drosophila* (black circle) frataxin in a pan-neural fashion (A), in the sensory organs and their precursors (B) or the glial cells (C) dramatically shortens the mean and maximum life span compared to control flies (white square). (D–F) Negative geotaxis experiment with 5- and 10-day-old individuals. Overexpression of frataxin in all 3 nervous system cell types strongly reduced the walking ability of the flies. The strongest effect was observed when the PNS driver (*neur*-GAL4) was applied. The statistical differences between the survival curves in A, B and C were analyzed using the Kapplan-Meier test, and both of the frataxins exhibited a statistically significant reduction (p<0.001) compared to that of the control individuals. The level of significance in D, E and F was determined using a one-way ANOVA with the *post hoc* Newman-Keuls test (* p<0.05). The error bars represent the standard error.

We further assessed whether human frataxin expression in neural tissues affects *Drosophila* fitness during adulthood. The length of the life span was examined using the *Appl* -GAL4, *neur* -GAL4 and *repo*-GAL4 drivers. The *FXN* flies exhibited a statistically significant decline in the mean (75%, 80% and 50%, respectively) and maximum life spans (74%, 75% and 56%, respectively). These decreases were larger than those that were observed after the overexpression of endogenous fly frataxin (Figure 2A–C).

To study the effect of frataxin overexpression on the nervous system functioning in *Drosophila*, the locomotor activity of the flies was analyzed. The overexpression of human or fly frataxin reduced the climbing ability of the flies in an age-dependent manner for all three of the drivers used. The larger reduction was observed for *FXN* with *neur-*GAL4 driver, which exhibited a 70% reduction in the 5-day-old flies and a 90% reduction in the 10-day-old flies (Figure 2E). *Appl* (Figure 2D) and *repo-*GAL4 (Figure 2F) also induced locomotor dysfunctions, although to a lesser extent (55% and 25% in 5-day-old flies, respectively). The findings indicate that aging appears to exacerbate the reduction in locomotor ability that results from frataxin overexpression.

**Figure 3:**
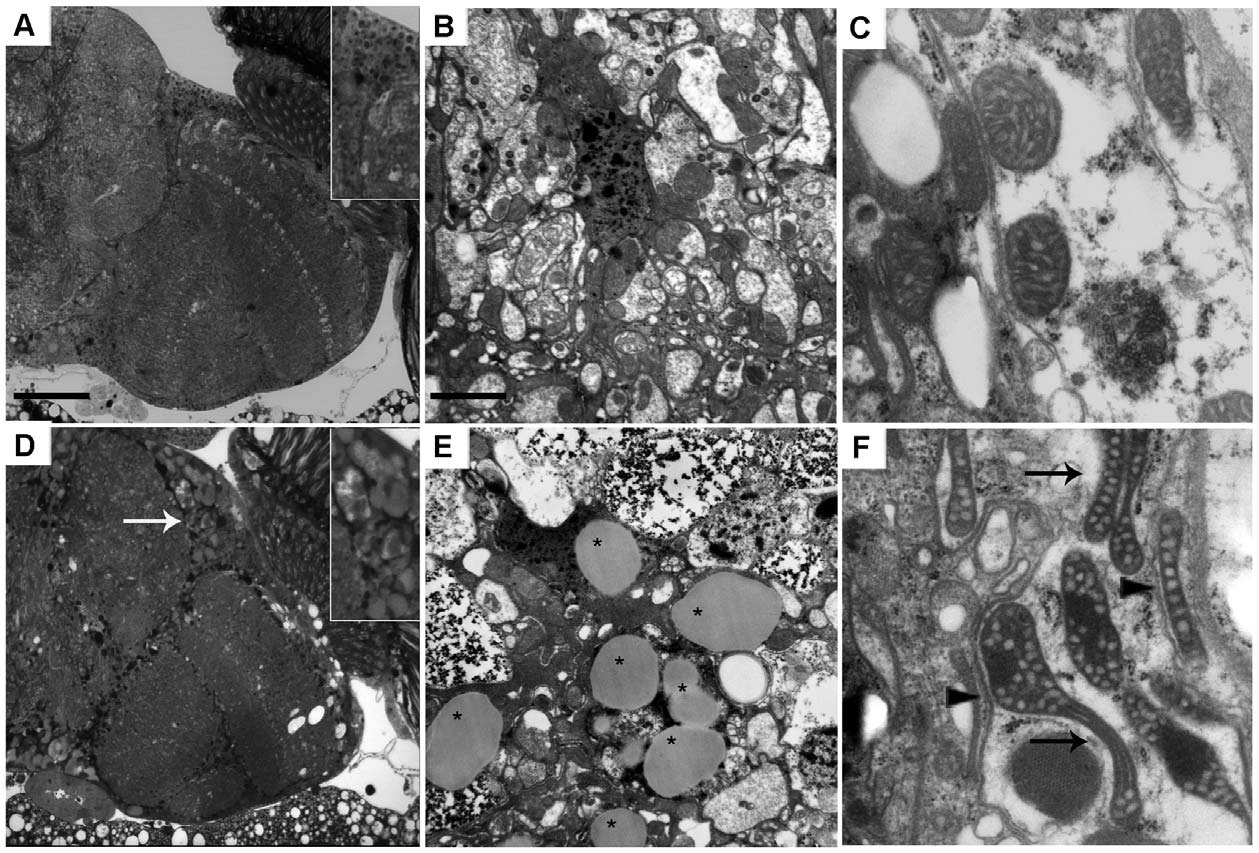
Strong degeneration and lipid droplet accumulation in glial cells overexpressing frataxin. (A–C) 25-day-old *Repo*-GAL4 / + controls; (D–F) 25-day-old *Repo*-GAL4 / UAS-*FXN*. (D) Overexpression of human frataxin induced a strong degeneration in the cortex (white arrow and 3X magnification box). (E, F) The electron microscopy analysis revealed an accumulation of lipid droplets (denoted by asterisks; E) in the glial cells of the frataxin-overexpressing brains and revealed mitochondria with altered morphologies (arrows; F) and internal vacuolization (arrow heads; F). The scale bar represents 50 µm (A, D) and 2.5 µm (B, C, E, F).

To identify the cellular pathology underlying the life span and locomotor phenotypes that have been associated with frataxin overexpression, the brain sections from flies that overexpressed human frataxin were analyzed using light and electron microscopy. The selective *FXN* expression in glial cells induced a strong age-related degeneration in the cortex and a neuropil vacuolization with the presence of droplet-like structures (Figure 3D). An ultrastructural analysis revealed a complete morphological disruption of the glial cells and the concomitant formation of lipid droplets (Figure 3E). Notably, a very similar phenotype was observed in the glial cells that lacked *Drosophila* frataxin [35]. Moreover, as shown in Figure 3F, several regions of the brain exhibited clear mitochondrial phenotypes, such as an abnormal morphology or vacuolization. Although the *Appl*-GAL4 > UAS-*FXN* flies exhibited a clear locomotor deficit and a shortened life span, these flies did not display brain abnormalities compared to the control age-matched individuals (data not shown).

These results indicate that an excess of frataxin impairs embryonic development and negatively affects fly fitness. Remarkably, our results from glial cells may suggest that frataxin overexpression alters cellular homeostasis in a similar manner to frataxin knock-down. These data indicate that a balance of frataxin levels is critical for the correct functioning of several cell types in the *Drosophila* nervous system.

### Overexpression of human frataxin enhances susceptibility to oxidative stress

**Figure 4:**
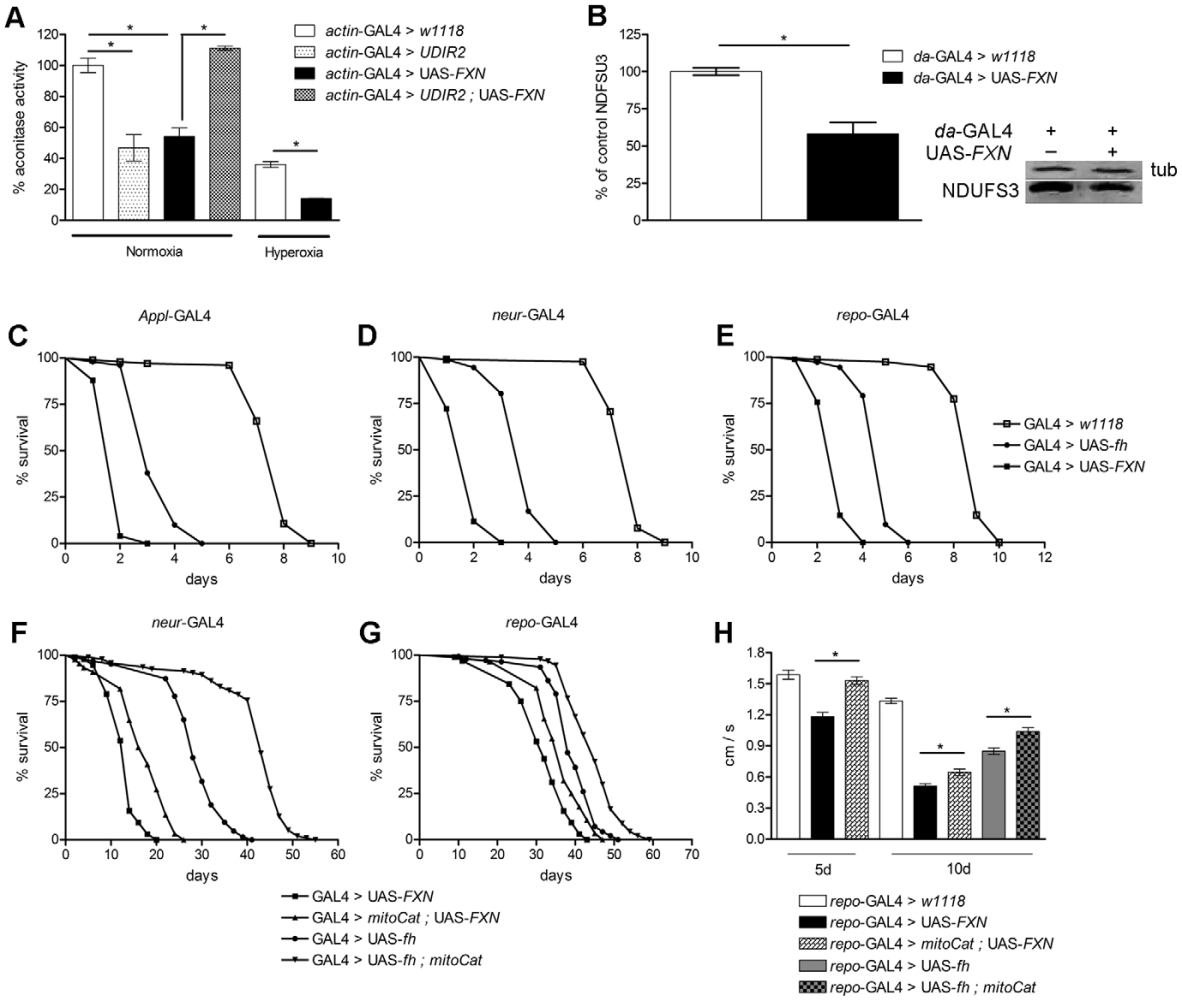
Molecular effects of frataxin overexpression and the involvement of oxidative stress. (A) The negative effects of human-frataxin overexpression on aconitase activity under normoxia and hyperoxia (99.5% O_2_) conditions. (B) Human frataxin overexpression triggered a reduction in the synthesis of the complex I subunit (amount normalized to the internal control α-tubulin). (C–E) Increased susceptibility to hyperoxia-mediated oxidative damage in flies overexpressing human and fly frataxin in the nervous system. (F,G) Constitutive expression of mitochondrial catalase (*mitoCat*) led to an extension of the mean and maximum life span of the flies with increased frataxin expression. This effect was strong in the peripheral nervous system (*neur*-GAL4) and moderate in the glial cells (*repo*-GAL4). (H) Co-expression of mitochondrial catalase (*mitoCat*) rescues (5d) and alleviates (10d) the locomotor deficits in the flies with an increased level of frataxin expression in the glial cells. The survival curves were analyzed using the Kapplan-Meier test. The level of significance in A, B and H was determined using a one-way ANOVA with a *post hoc* Newman-Keuls test (*p<0.05). The error bars represent the standard error.

One of the most characteristic biochemical defects that is associated with a loss of frataxin is the reduction of aconitase activity [18], [19], [31]. Therefore, we tested whether the overexpression of human frataxin in *Drosophila* would also affect the activity of this enzyme. Aconitase activity was measured in *actin*-GAL4>UAS-*FXN* L3 larvae because the ubiquitous expression of *FXN* caused lethality before adult eclosion (Table 1). Notably, these larvae exhibited a 50% reduction in aconitase activity under normoxia conditions (Figure 4A).

Several FA models have also resulted in a strong reduction of other Fe-S-cluster-containing proteins, such as the complex I subunit, SdhA, SdhB or the Rieske protein [52], [53]. Thus, we examined whether frataxin overexpression would produce a similar effect on the NDUFS3 levels. In agreement with the results of other studies [52], [53], we observed that FXN overexpression triggered a 40% reduction in the amount of NDUFS3 protein expression (Figure 4B) without a change in the mRNA levels (data not shown), which excludes the possibility of a transcriptional regulatory mechanism. The reduction of the aconitase activity and the decrease in the amount of NDUFS3 expression are direct evidence of a reduction in the Fe-S cluster formation in our overexpression model.

Given that aconitase is a specific target of oxidative stress [54], [55], we assessed the functional integrity of aconitase in *FXN* larvae after oxidative stress injury. As expected, the ubiquitous expression of *FXN,* combined with a hyperoxia treatment, also resulted in a two-fold reduction in aconitase activity compared to hyperoxia-treated controls (Figure 4A). Moreover, aconitase was seriously affected in flies with general *fh* overexpression that was combined with hyperoxia [31].

To test whether oxidative stress was involved in the phenotypes that were observed in the nervous system, we exposed flies that overexpressed *FXN* to a highly oxidative atmosphere (99.5% O_2_). Under these conditions, we observed a strong decrease in the mean (65%) and maximum life span (50%) when compared to those of the controls (Figure 4C–E). Again, the *FXN* flies displayed stronger phenotypes than the flies that overexpressed *fh*.

In *Drosophila*, a constitutive increase in the mitochondrial-driven catalase (*mitoCat*) activity is known to improve the resistance to oxidative damage [56]. Moreover, the expression of this enzyme has been reported to extend the life span of frataxin-deficient flies and to improve the resistance of these flies to oxidative insult [32]. Therefore, we examined the effect of this free radical scavenger on life span and locomotor performance in our frataxin overexpressing flies. As illustrated in Figure 4F, *mitoCat* produced a significant prolongation of life span when frataxin was overexpressed using *neur*-GAL4 and caused a statistically non-significant increase for repo-GAL4 (Figure 4G). In addition, *mitoCat* ameliorated the climbing deficiency that was induced by *FXN* or *fh* overexpression in glial cells (Figure 4H).

These results clearly identify oxidative stress and mainly hydrogen peroxides as key factors in the frataxin overexpression phenotypes that have been observed.

### Overexpressed FXN do not form aggregates or misfold in *Drosophila*

The overexpression of human or *Drosophila* frataxins produce a phenotype that is surprisingly similar to the phenotype that is observed in frataxin-depleted mutants. Thus, we assessed whether the overexpression of frataxin would induce neomorph phenotypes and lead to a loss of function phenocopy *via* protein aggregation or misfolding.

Heat shock proteins have been reported to display rescuing effects in *Drosophila* neurodegenerative models of protein misfolding or aggregation [57]–[59]. Therefore, we assessed whether the co-expression of heat-shock proteins would lead to beneficial effects in our frataxin overload scenarios. Human heat-shock cognates were used in combination with *FXN,* and *Drosophila Hsp70* and *Hsp22* were co-expressed with *fh*; however, these heat-shock proteins were not able to improve the climbing performance of the frataxin-overexpressing flies (data not shown).

**Figure 5:**
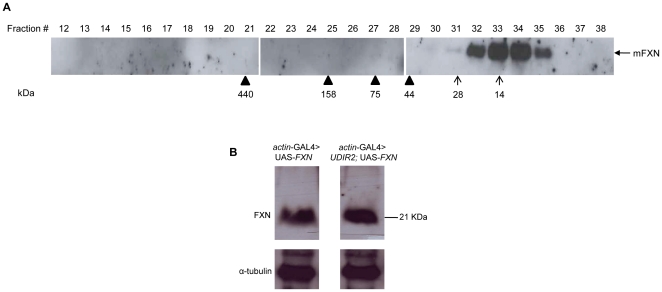
FXN does not form aggregates in *Drosophila,* and its expression is not diluted when it is coexpressed with the interference of *fh*. (A) Mitochondrial cell extracts were obtained from *actin*-GAL4>UAS-*FXN* larvae and were size fractionated. The fractions were subsequently analyzed using SDS-PAGE and western blotting with an anti-human frataxin antibody. The positions of ferritin (440 kDa), aldolase (158 kDa), conalbumin (75 kDa), and ovalbumin (44 kDa) and the estimated position for the frataxin monomer and dimer are indicated as arrowheads and arrows, respectively. (B) Detection of FXN protein in *actin*-GAL4>UAS-*FXN* and *actin*-GAL4>*UDIR2*; UAS-*FXN* larvae. The FXN protein is not diluted when it is co-expressed with an RNA interference construct of *fh*. α-tubulin was used as a loading control.

Size exclusion chromatography was conducted for the mitochondrial matrix proteins from the *FXN*-overexpressing larvae, and the fractions that were obtained were analyzed using western blotting for the presence of *FXN*. In our experiments, after the overexpression of *FXN* in *Drosophila,* human frataxin was recovered as a monomeric form (Figure 5A), and no high molecular weight frataxin aggregates (dimers, trimers or multimeric forms of frataxin) were detected in the void volume (fraction 14). It was nevertheless possible that frataxin protein aggregates were not solubilized with the mitochondrial matrix proteins. Insoluble cellular proteins were solubilized, and the western blot did not reveal the presence of frataxin in this solubilized fraction (data not shown).

Collectively, these results indicate that frataxin aggregation or misfolding is unlikely to be the mechanism behind the phenotypes that have been observed.

### *FXN* overexpression restores the aconitase activity in *fh* deficient individuals

In terms of their sequences and other structural properties, the degree of conservation between FH and FXN [33], [60] indicates a possible similarity in the function of these proteins. In support of this hypothesis, we have shown that *Drosophila* and human frataxin overexpression produce similar phenotypes. Lastly, we investigated the consequences of expressing *FXN* in *fh*-knockdown flies. To accomplish this task, we generated flies by combining *FXN* and *UDIR2* transgenes, and the latter transgene induced a 90% reduction in the level of *fh* expression [34].

A decrease in the level of aconitase activity appears to be the most sensitive biological and biochemical marker in the FA fly models [31]–[35]. As a result, we tested whether aconitase could be rescued in *fh* knockdown flies expressing *FXN*. In agreement with previously published results [34], the depletion of fly frataxin led to a two-fold reduction in aconitase activity in larvae compared to controls. Remarkably, the expression of *FXN* prevented aconitase inactivation, and the aconitase activity was recovered to levels that were comparable with those of the controls (Figure 4A).

Considering the possibility that the rescue of the aconitase activity was related to degradation of the *FXN* mRNA that was induced by the RNAi transgene directed to *fh*, we assessed the human frataxin protein levels using western blotting. Comparable levels of FXN were observed in the *actin*-GAL4>UAS-*FXN* and *actin*-GAL4>UAS-*UDIR2*;UAS-*FXN* larvae (Figure 4B).

We can conclude that human frataxin is able to replace the endogenous *Drosophila* frataxin, which suggests that these proteins play an equivalent role in the cell biology of these organisms.

## Discussion

Friedreich's ataxia is the most common autosomal recessive ataxia in the Caucasian population. This disease exhibits an irreversible progression that confines a patient to a wheelchair and leads to an early death. Moreover, although different treatments are currently being developed and assessed in clinical trials, there is no cure available. To generate effective and adequate therapies for Friedreich's ataxia, it is imperative to define the function of the frataxin protein. Unfortunately, the precise function of this protein is still a matter of debate. Although overexpression studies do not represent a disease model for FA, these studies are a useful approach to decipher the mechanism of action of frataxin. Furthermore, these models may provide insight into the effects of an excess of frataxin, which is a critical factor for the validation of treatments that are based on an increase in this protein's expression.

Frataxins are a highly conserved family of proteins. *In silico* analyses have shown that *Drosophila* frataxin and frataxin proteins that are found in other species share large percentages of identity and similarity in their sequence and a common secondary structure [33]. The closest match between the human and the fly frataxins involves a stretch of 38 amino acids at the C-terminus, which is encoded by *fh* exon 2, and exons 4 and 5a of the *FXN* gene, respectively. This highly conserved region is very likely to form a functional domain with a β-sheet structure that is flanked by α-helices, where the sequence is less conserved [33]. Moreover, *Drosophila* frataxin has similar biophysical properties to human frataxin [60] and exhibits a mitochondrial localization [31]. In agreement with mouse models of FA [19], a strong systemic depletion of *fh* induces lethality during early development [31], [34], whereas the moderate reduction of *fh* produces phenotypes that parallel the symptoms of FA patients [31]. In addition, the tissue-specific silencing of frataxin leads to the mimicking of human phenotypes [31], [34]–[36]. Collectively, these data indicate that these proteins may be playing identical roles; however, their functional equivalence had not yet been demonstrated experimentally.

In the present work, we generated a *Drosophila* strain that overexpressed the human frataxin (*FXN*). Flies with an increased level of expression of *FXN* exhibited similar defects to those found after endogenous frataxin overexpression, such as an alteration of development, a reduction in viability, life span and motor ability [31] and clear manifestations of nervous system degeneration, impaired Fe-S cluster formation and an enhanced susceptibility to oxidative stress. These results support the hypothesis that these frataxins are functionally equivalent. In the present work, we have shown that *FXN* was able to recover aconitase activity, the most sensitive biochemical marker of FA, in frataxin-deficient larvae.

Although the effect of frataxin overproduction has been investigated in several models, our studies using *Drosophila* as a model are the only studies to show deleterious defects. Human frataxin overexpression has been reported to be innocuous in mice [43] or to stimulate energy production [17]. Frataxin promotes OXPHOS activation in cell culture [15], [45] and increases cellular antioxidant defense in cell culture and yeast [44], [47]. In agreement with these findings, the overexpression of frataxin in *Drosophila* that was conducted by Runko *et al.* [46] increased the resistance to oxidative stress and extended the life span of the flies. These differences may be related to the characteristics of the models but may also result from quantitative differences in the level of frataxin overproduction. A closer look at the data from these studies reveals that a slight overexpression of frataxin was reported, from 2 to 6 times that of baseline, depending on the model and/or the tissue that was used. Conversely, in the present work, and in Llorens *et al.* [31], a minimum of 9-fold increase in frataxin production was induced. Therefore, it is possible that moderate overproduction of this protein may lead to beneficial effects, whereas the expression of frataxin beyond a given threshold may have multiple effects inducing toxicity. Taken together, these results indicate that frataxin requires an optimal balance of its expression level to carry out its function properly. To date, very little is known about the regulation of frataxin expression. It has been reported that the transcription factors *Hypoxia-inducible Factor 2alpha* [61], SRF and TFAP2 [62] are involved in regulating the expression of frataxin, as may be the iron content of the cell [63]. Analyzing the mechanism of this regulatory network is a new field that will provide new targets for future therapies.

In agreement with the results of the present study, the overproduction of yeast frataxin has been shown to impair Fe-S cluster formation and to lead to a reduction in aconitase and SDH activities [47]. These authors proposed that the trimeric form of frataxin may be responsible for the interaction with the complex of iron-sulfur cluster machinery. Thus, the increase in frataxin oligomerization due to its overexpression would lower the amounts of trimers that restrict the production of the cluster. To clarify the toxic mechanism that is responsible for frataxin overexpression in *Drosophila*, we considered the possibility that FXN was also inducing protein aggregation or misfolding, leading to a reduction in the level of functional frataxin. However, the results of our gel filtration assays did not show any shift in FXN to higher molecular masses, and the protein was recovered in the monomeric form. In addition, the overexpression of human or fly heat-shock proteins did not lead to any improvement in the frataxin overexpression phenotypes. These results indicate that protein aggregation and misfolding are not the central factors leading to the frataxin overexpression defects. Notably, our results do not reproduce the data from the experiments using bacterial [64], [65] or yeast [47], [66] models in which frataxin forms multimers. These findings indicate that oligomerization does not occur in *Drosophila*. Our results are in agreement with the findings of Kondapalli *et al.* [60], who reported that FH seems to be less prone to aggregation *in vitro* than the yeast protein, which appeared as a monomer in most of the conditions that were tested.

In our overexpression model, we observed aconitase inactivation and a reduction in the NDUFS3 levels, indicating an alteration in Fe-S cluster formation. These results argue in favor of the role of frataxin as an inhibitor of the Fe-S cluster assembly machinery, as previously suggested by Adinolfi *et al*. [24]. However, this inhibitory function is not consistent with the stimulation of the respiratory chain that has been described by other authors [15], [17], [45]. Therefore, we propose that a moderate overexpression of frataxin may promote the synthesis of clusters or promote their stabilization when they are incorporated into apo-proteins, as has been previously suggested [16], [28]. In contrast, a larger increase in the overexpression of frataxin may lead to a reduction in Fe-S cluster formation, regardless of this proteins function as an activator [12], [18]–[23] or suppressor [24], by saturating the ISC machinery or by sequestering the proteins from the machinery that interact with frataxin. Frataxin has been recently proposed to maintain the ISCU/NFS1/ISD11 interaction [23]; however, an increase of frataxin may over-stabilize this complex and cause an Fe-S cluster deficiency.

Our results that show the constitutive expression of mitochondrial catalase demonstrate that oxidative stress and hydrogen peroxides are key factors in the frataxin overexpression phenotypes. Notably, the positive effect of mitochondrial catalase in counteracting frataxin defects has been previously reported [32]. These results suggest a common mechanism in the loss-of-function and gain-of-function phenotypes that are induced by frataxin. Remarkably, in Llorens *et al.* [31] and in the present study, other similar phenotypes between strong frataxin overproduction and frataxin depletion have been described, such as structural defects that lead to a reduction in longevity and locomotor capabilities and an increased sensitivity to oxidative damage. Moreover, the overexpression of *FXN* in glial cells leads to the presence of the lipid droplets and the brain degeneration that have been exhibited by glial-frataxin deficient flies [35].

In conclusion, we demonstrate that overexpression of *Drosophila* and human frataxins induces severe developmental problems in flies, a shortening of life span, brain degeneration and reduced aconitase activity. Moreover, the control of frataxin expression emerges as a crucial element for present and future treatments, such as gene therapy approaches, aimed at increasing frataxin levels.

## References

[1] Palau F, Espinós C (2006). Autosomal recessive cerebellar ataxias.. Orphanet J Rare Dis.

[2] Campuzano V, Montermini L, Moltò MD, Pianese L, Cossée M (1996). Friedreich's ataxia: autosomal recessive disease caused by an intronic GAA triplet repeat expansion.. Science.

[3] Ohshima K, Montermini L, Wells RD, Pandolfo M (1998). Inhibitory effects of expanded GAA.TTC triplet repeats from intron I of the Friedreich ataxia gene on transcription and replication in vivo. J. Biol.. Chem.

[4] Sakamoto N, Chastain PD, Parniewski P, Ohshima K, Pandolfo M (1999). Sticky DNA: self-association properties of long GAA.TTC repeats in R.R.Y triplex structures from Friedreich's ataxia. Mol.. Cell.

[5] Campuzano V, Montermini L, Lutz Y, Cova L, Hindelang C (1997). Frataxin is reduced in Friedreich ataxia patients and is associated with mitochondrial membranes. Hum. Mol.. Genet.

[6] Harding AE (1981). Friedreich's ataxia: a clinical and genetic study of 90 families with an analysis of early diagnostic criteria and intrafamilial clustering of clinical features.. Brain.

[7] Harding AE (1993). Clinical features and classification of inherited ataxias.. Adv Neurol.

[8] Harding AE, Hewer RL (1983). The heart disease of Friedreich's ataxia: a clinical and electrocardiographic study of 115 patients, with an analysis of serial electrocardiographic changes in 30 cases.. Q J Med.

[9] Gibson TJ, Koonin EV, Musco G, Pastore A, Bork P (1996). Friedreich's ataxia protein: phylogenetic evidence for mitochondrial dysfunction.. Trends Neurosci.

[10] Babcock M, de Silva D, Oaks R, Davis-Kaplan S, Jiralerspong S (1997). Regulation of mitochondrial iron accumulation by Yfh1p, a putative homolog of frataxin.. Science.

[11] Cavadini P, Gellera C, Patel PI, Isaya G (2000). Human frataxin maintains mitochondrial iron homeostasis in Saccharomyces cerevisiae. Hum. Mol.. Genet.

[12] Mühlenhoff U, Gerber J, Richhardt N, Lill R (2003). Components involved in assembly and dislocation of iron-sulfur clusters on the scaffold protein Isu1p.. EMBO J.

[13] Park S, Gakh O, O'Neill HA, Mangravita A, Nichol H (2003). Yeast frataxin sequentially chaperones and stores iron by coupling protein assembly with iron oxidation. J. Biol.. Chem.

[14] Moreno-Cermeño A, Obis E, Bellí G, Cabiscol E, Ros J (2010). Frataxin Depletion in Yeast Triggers Up-regulation of Iron Transport Systems before Affecting Iron-Sulfur Enzyme Activities. J. Biol.. Chem.

[15] Ristow M, Pfister MF, Yee AJ, Schubert M, Michael L (2000). Frataxin activates mitochondrial energy conversion and oxidative phosphorylation. Proc.. Natl Acad Sci U S A.

[16] González-Cabo P, Vázquez-Manrique RP, García-Gimeno MA, Sanz P, Palau F (2005). Frataxin interacts functionally with mitochondrial electron transport chain proteins. Hum. Mol.. Genet.

[17] Schulz TJ, Westermann D, Isken F, Voigt A, Laube B (2010). Activation of mitochondrial energy metabolism protects against cardiac failure.. Aging (Albany NY).

[18] Rötig A, de Lonlay P, Chretien D, Foury F, Koenig M (1997). Aconitase and mitochondrial iron-sulphur protein deficiency in Friedreich ataxia. Nat.. Genet.

[19] Puccio H, Simon D, Cossée M, Criqui-Filipe P, Tiziano F (2001). Mouse models for Friedreich ataxia exhibit cardiomyopathy, sensory nerve defect and Fe-S enzyme deficiency followed by intramitochondrial iron deposits. Nat.. Genet.

[20] Ramazzotti A, Vanmansart V, Foury F (2004). Mitochondrial functional interactions between frataxin and Isu1p, the iron-sulfur cluster scaffold protein, in Saccharomyces cerevisiae.. FEBS Lett.

[21] Vivas E, Skovran E, Downs DM (2006). Salmonella enterica strains lacking the frataxin homolog CyaY show defects in Fe-S cluster metabolism in vivo.. J Bacteriol.

[22] Stemmler TL, Lesuisse E, Pain D, Dancis A (2010). Frataxin and mitochondrial FeS cluster biogenesis. J. Biol.. Chem.

[23] Schmucker S, Martelli A, Colin F, Page A, Wattenhofer-Donzé M (2011). Mammalian Frataxin: An Essential Function for Cellular Viability through an Interaction with a Preformed ISCU/NFS1/ISD11 Iron-Sulfur Assembly Complex.. PLoS ONE.

[24] Adinolfi S, Iannuzzi C, Prischi F, Pastore C, Iametti S (2009). Bacterial frataxin CyaY is the gatekeeper of iron-sulfur cluster formation catalyzed by IscS. Nat. Struct. Mol.. Biol.

[25] Emond M, Lepage G, Vanasse M, Pandolfo M (2000). Increased levels of plasma malondialdehyde in Friedreich ataxia.. Neurology.

[26] Schulz JB, Dehmer T, Schöls L, Mende H, Hardt C (2000). Oxidative stress in patients with Friedreich ataxia.. Neurology.

[27] Ristow M, Mulder H, Pomplun D, Schulz TJ, Müller-Schmehl K (2003). Frataxin deficiency in pancreatic islets causes diabetes due to loss of beta cell mass. J. Clin.. Invest.

[28] Bulteau A, O'Neill HA, Kennedy MC, Ikeda-Saito M, Isaya G (2004). Frataxin acts as an iron chaperone protein to modulate mitochondrial aconitase activity.. Science.

[29] Bulteau A, Dancis A, Gareil M, Montagne J, Camadro J (2007). Oxidative stress and protease dysfunction in the yeast model of Friedreich ataxia. Free Radic. Biol.. Med.

[30] Vázquez-Manrique RP, González-Cabo P, Ros S, Aziz H, Baylis HA (2006). Reduction of Caenorhabditis elegans frataxin increases sensitivity to oxidative stress, reduces lifespan, and causes lethality in a mitochondrial complex II mutant.. FASEB J.

[31] Llorens JV, Navarro JA, Martínez-Sebastián MJ, Baylies MK, Schneuwly S (2007). Causative role of oxidative stress in a Drosophila model of Friedreich ataxia.. FASEB J.

[32] Anderson PR, Kirby K, Orr WC, Hilliker AJ, Phillips JP (2008). Hydrogen peroxide scavenging rescues frataxin deficiency in a Drosophila model of Friedreich's ataxia. Proc.. Natl Acad Sci U S A.

[33] Cañizares J, Blanca JM, Navarro JA, Monrós E, Palau F (2000). dfh is a Drosophila homolog of the Friedreich's ataxia disease gene.. Gene.

[34] Anderson PR, Kirby K, Hilliker AJ, Phillips JP (2005). RNAi-mediated suppression of the mitochondrial iron chaperone, frataxin, in Drosophila. Hum. Mol.. Genet.

[35] Navarro JA, Ohmann E, Sanchez D, Botella JA, Liebisch G (2010). Altered lipid metabolism in a Drosophila model of Friedreich's ataxia. Hum. Mol.. Genet.

[36] Shidara Y, Hollenbeck PJ (2010). Defects in mitochondrial axonal transport and membrane potential without increased reactive oxygen species production in a Drosophila model of Friedreich ataxia.. J Neurosci.

[37] Finelli A, Kelkar A, Song H, Yang H, Konsolaki M (2004). A model for studying Alzheimer's Abeta42-induced toxicity in Drosophila melanogaster. Mol. Cell.. Neurosci.

[38] Marsh JL, Pallos J, Thompson LM (2003). Fly models of Huntington's disease. Hum. Mol.. Genet 12 Spec No.

[39] Feany MB, Bender WW (2000). A Drosophila model of Parkinson's disease.. Nature.

[40] Venderova K, Kabbach G, Abdel-Messih E, Zhang Y, Parks RJ (2009). Leucine-Rich Repeat Kinase 2 interacts with Parkin, DJ-1 and PINK-1 in a Drosophila melanogaster model of Parkinson's disease. Hum. Mol.. Genet.

[41] Fernandez-Funez P, Nino-Rosales ML, de Gouyon B, She WC, Luchak JM (2000). Identification of genes that modify ataxin-1-induced neurodegeneration.. Nature.

[42] Mutsuddi M, Marshall CM, Benzow KA, Koob MD, Rebay I (2004). The spinocerebellar ataxia 8 noncoding RNA causes neurodegeneration and associates with staufen in Drosophila. Curr.. Biol.

[43] Miranda CJ, Santos MM, Ohshima K, Tessaro M, Sequeiros J (2004). Frataxin overexpressing mice.. FEBS Lett.

[44] Shoichet SA, Bäumer AT, Stamenkovic D, Sauer H, Pfeiffer AFH (2002). Frataxin promotes antioxidant defense in a thiol-dependent manner resulting in diminished malignant transformation in vitro. Hum. Mol.. Genet.

[45] Schulz TJ, Thierbach R, Voigt A, Drewes G, Mietzner B (2006). Induction of oxidative metabolism by mitochondrial frataxin inhibits cancer growth: Otto Warburg revisited. J. Biol.. Chem.

[46] Runko AP, Griswold AJ, Min K (2008). Overexpression of frataxin in the mitochondria increases resistance to oxidative stress and extends lifespan in Drosophila.. FEBS Lett.

[47] Seguin A, Bayot A, Dancis A, Rogowska-Wrzesinska A, Auchère F (2009). Overexpression of the yeast frataxin homolog (Yfh1): contrasting effects on iron-sulfur cluster assembly, heme synthesis and resistance to oxidative stress.. Mitochondrion.

[48] Rubin GM, Spradling AC (1982). Genetic transformation of Drosophila with transposable element vectors.. Science.

[49] Kirby K, Hu J, Hilliker AJ, Phillips JP (2002). RNA interference-mediated silencing of Sod2 in Drosophila leads to early adult-onset mortality and elevated endogenous oxidative stress.. Proc Natl Acad Sci U S A.

[50] Patel NH (1994). Imaging neuronal subsets and other cell types in whole-mount Drosophila embryos and larvae using antibody probes.. Methods Cell Biol.

[51] Botella JA, Ulschmid JK, Gruenewald C, Moehle C, Kretzschmar D (2004). The Drosophila carbonyl reductase sniffer prevents oxidative stress-induced neurodegeneration. Curr.. Biol.

[52] Sutak R, Xu X, Whitnall M, Kashem MA, Vyoral D (2008). Proteomic analysis of hearts from frataxin knockout mice: marked rearrangement of energy metabolism, a response to cellular stress and altered expression of proteins involved in cell structure, motility and metabolism.. Proteomics.

[53] Guillon B, Bulteau A, Wattenhofer-Donzé M, Schmucker S, Friguet B (2009). Frataxin deficiency causes upregulation of mitochondrial Lon and ClpP proteases and severe loss of mitochondrial Fe-S proteins.. FEBS J.

[54] Das N, Levine RL, Orr WC, Sohal RS (2001). Selectivity of protein oxidative damage during aging in Drosophila melanogaster.. Biochem J.

[55] Delaval E, Perichon M, Friguet B (2004). Age-related impairment of mitochondrial matrix aconitase and ATP-stimulated protease in rat liver and heart. Eur.. J Biochem.

[56] Orr WC, Sohal RS (1992). The effects of catalase gene overexpression on life span and resistance to oxidative stress in transgenic Drosophila melanogaster. Arch. Biochem.. Biophys.

[57] Warrick JM, Chan HY, Gray-Board GL, Chai Y, Paulson HL (1999). Suppression of polyglutamine-mediated neurodegeneration in Drosophila by the molecular chaperone HSP70. Nat.. Genet.

[58] Auluck PK, Chan HYE, Trojanowski JQ, Lee VMY, Bonini NM (2002). Chaperone suppression of alpha-synuclein toxicity in a Drosophila model for Parkinson's disease.. Science.

[59] Fernandez-Funez P, Casas-Tinto S, Zhang Y, Gómez-Velazquez M, Morales-Garza MA (2009). In vivo generation of neurotoxic prion protein: role for hsp70 in accumulation of misfolded isoforms.. PLoS Genet.

[60] Kondapalli KC, Kok NM, Dancis A, Stemmler TL (2008). Drosophila frataxin: an iron chaperone during cellular Fe-S cluster bioassembly.. Biochemistry.

[61] Oktay Y, Dioum E, Matsuzaki S, Ding K, Yan L (2007). Hypoxia-inducible factor 2alpha regulates expression of the mitochondrial aconitase chaperone protein frataxin. J. Biol.. Chem.

[62] Li K, Singh A, Crooks DR, Dai X, Cong Z (2010). Expression of human frataxin is regulated by transcription factors SRF and TFAP2.. PLoS ONE.

[63] Li K, Besse EK, Ha D, Kovtunovych G, Rouault TA (2008). Iron-dependent regulation of frataxin expression: implications for treatment of Friedreich ataxia. Hum. Mol.. Genet.

[64] Bou-Abdallah F, Adinolfi S, Pastore A, Laue TM, Dennis Chasteen N (2004). Iron binding and oxidation kinetics in frataxin CyaY of Escherichia coli. J. Mol.. Biol.

[65] Layer G, Ollagnier-de Choudens S, Sanakis Y, Fontecave M (2006). Iron-sulfur cluster biosynthesis: characterization of Escherichia coli CYaY as an iron donor for the assembly of [2Fe-2S] clusters in the scaffold IscU. J. Biol.. Chem.

[66] Aloria K, Schilke B, Andrew A, Craig EA (2004). Iron-induced oligomerization of yeast frataxin homologue Yfh1 is dispensable in vivo.. EMBO Rep.

